# Reagentless Electrochemical Detection of Tumor Biomarker Based on Stable Confinement of Electrochemical Probe in Bipolar Silica Nanochannel Film

**DOI:** 10.3390/nano13101645

**Published:** 2023-05-15

**Authors:** Xile Zhou, Qianqian Han, Jinming Zhou, Chaoxu Liu, Jiyang Liu

**Affiliations:** 1 Department of Colorectal Surgery, The First Affiliated Hospital, College of Medicine, Zhejiang University, Hangzhou 310003, China; 1520003@zju.edu.cn; 2 Department of Chemistry, Zhejiang Sci-Tech University, Hangzhou 310018, China; 202130107292@mails.zstu.edu.cn; 3 Drug Development and Innovation Center, College of Chemistry and Life Sciences, Zhejiang Normal University, 688 Yingbin Road, Jinhua 321004, China; zhoujinming@zjnu.edu.cn

**Keywords:** electrochemical sensor, reagentless, probe-integrated, silica nanochannel array, carcinoembryonic antigen

## Abstract

The development of simple and probe-integrated aptamer sensors for the electrochemical detection of tumor biomarkers is of great significance for the diagnosis of tumors and evaluation of prognosis. In this work, a probe-integrated aptamer sensor is demonstrated based on the stable confinement of an electrochemical probe in a bipolar nanochannel film, which can realize the reagentless electrochemical detection of the tumor biomarker carcinoembryonic antigen (CEA). To realize the stable immobilization of a large amount of the cationic electrochemical probe methylene blue (MB), a two-layer silica nanochannel array (SNF) with asymmetric charge was grown on the supporting electrode from bipolar SNF (bp-SNF). The inner SNF is negatively charged (n-SNF), and the outer-layer SNF is positively charged (p-SNF). The dual electrostatic interaction including the electrostatic adsorption from n-SNF and the electrostatic repulsion from p-SNF achieve the stable confinement of MB in bp-SNF. The recognitive interface is fabricated by the covalent immobilization of the CEA aptamer on the outer surface of bp-SNF, followed by the blocking of non-specific binding sites. Owing to the stable and abundant immobilized probes and highly specific aptamer interface, the developed aptamer sensor enables the sensitive detection of CEA in the range of 1 pg/mL to 1 μg/mL with a low limit of detection (LOD, 0.22 pg/mL, S/N = 3). Owing to the high selectivity and stability of the developed biosensor, reagentless electrochemical detection of CEA in serum was realized.

## 1. Introduction

Cancer gravely endangers human health. The key to cancer prevention and treatment lies in early diagnosis and treatment. However, there are still significant challenges in the early diagnosis of cancer [1]. For example, traditional detection methods such as X-ray, computed tomography (CT), and ultrasound can only detect the shape and size of tumors [2]. With the in-depth development of molecular biology and immunology research, tumor biomarkers have been proven to be important indicators in the clinical diagnosis of cancer, and their appearance often precedes morphological or biological changes in cells or tissue. Therefore, the detection of tumor markers with high sensitivity and selectivity is of great significance for the early and accurate diagnosis and treatment of cancer [3,4]. Carcinoembryonic antigen (CEA) is one of the most common tumor biomarkers, which has been widely used in the clinical treatment of various malignant tumors, such as colorectal cancer [5], breast cancer [6], lung cancer [7], and so on. It also has a unique value in monitoring the recurrence and metastasis of colorectal cancer [8]. Briefly, CEA is a polysaccharide protein complex with a molecular weight of 180 to 200 kDa, which belongs to the structural antigen on the surface of tumor cells [9]. CEA can be detected in various bodily fluids because it is formed in the cytoplasm and then passes through the cell membrane and enters the surrounding bodily fluid. Thus, the detection of CEA in serum is important in the diagnosis of tumors and evaluation of prognosis.

The traditional method for CEA determination is via immunoassay, which is based on the specificity and sensitivity between antibody and antigen [10,11,12,13]. The existing CEA detection method mostly uses monoclonal antibodies, which involve complex and time-consuming preparation, high prices, and poor stability [14]. With the recent development of molecular biology technology, nucleic acid aptamers have received widespread attention. Aptamers are oligonucleotides selected using the systematic evolution of ligands by the exponential enrichment (SELEX) technique [15]. Their targets are very broad, including small molecules, proteins, cells, and even tissues [16,17]. Compared to an antibody, an aptamer has many advantages, such as small molecular weight, high affinity and specificity for targets, ease of chemical synthesis, stability, and ease of modification [17,18]. Currently, detection commonly uses fluorescence [19], electrochemiluminescence [20], electrochemistry [21], etc. Among those, electrochemical technology has attracted significant attention mainly due to its outstanding advantages, such as simple instruments; low cost; fast response; and the ability to achieve miniaturized, integrated, and automated detection [22,23,24,25,26]. Until now, electrochemical CEA antigen aptasensors with different signal amplifications (e.g., catalytic recycling of DNase I, exonuclease III, and hybrid chain reaction) have been reported [27,28,29,30]. Compared with the sandwich detection mode, CEA detection by measuring the signal changes of the electrochemical probe before and after the binding of CEA to a recognitive aptamer is simpler. This includes two detection strategies. One is to add a free electrochemical probe to the solution, and the other is to immobilize the probe on the electrode surface. Compared to the former, probe-integrated electrochemical detection has the advantage of simple operation and no interference with the test solution, leading to reagentless detection. Therefore, the development of simple and probe-integrated aptamer sensors for electrochemical detection of CEA is highly desirable. 

The microstructure of the electrode surface can significantly increase the amount of fixed electrochemical probes and construct biological recognition interfaces [31,32]. The growth of a silica nanochannel array (SNF) perpendicular to the underlying electrode is effective for preparing electrodes with a microstructure [33,34,35]. As an ultrathin nanofilm with an adjustable thickness between 20 and 200 nm, SNF has a uniform nanochannel (typically 2 to 3 nm in diameter) with a high density (up to 75,000 pores/μm^2^) [36]. Currently, it is possible to achieve convenient, low-cost, and large-area (e.g., tens of square centimeters) growth of SNF on the surface of an electrode [37]. In addition to excellent chemical/thermal stability and good biocompatibility, SNF has a rigid and three-dimensional (3D) monolith structure [38]. On the one hand, the rigid monolithic silica structure of SNF experiences no significant swelling during use, which can significantly improve the stability and reproducibility of the constructed sensor. On the other hand, the inner surfaces of the nanochannel and the outer surface of the film can serve as independent modification domains [39,40]. For instance, an electrochemical probe can be immobilized within the nanochannel while recognized ligands can be introduced on the outer surface of the film [41]. More importantly, SNF nanochannels have screening capabilities based on the size and electrostatic characteristics of molecules due to the ultrasmall diameter close to the Debye length. Thus, macromolecules (e.g., protein and DNA) or large particles cannot enter SNF nanochannels, leading to outstanding anti-fouling and anti-interference performance [42,43]. At the same time, the negatively charged surface obtained from the dissociation of silanol groups (Si-OH, p*K*_a_ ~ 2) on the surface of SNF can significantly enrich cations by electrostatic charge [44,45]. Therefore, SNF exhibits great potential for the construction of probe-integrated aptamer sensors for the electrochemical detection of CEA. 

In this work, a probe-integrated aptamer sensor was fabricated using SNF as the structural block, which can realize sensitive electrochemical detection of CEA. A bipolar SNF (bp-SNF) film with asymmetric surface charges was prepared by successively growing a bilayer SNF on the electrode surface. The inner SNF is negatively charged (n-SNF), and the outer SNF is positively charged from introducing amino groups on the surface (p-SNF). Owing to the strong electrostatic adsorption from n-SNF, the cationic electrochemical probe methylene blue (MB) can be significantly enriched. This electrostatic adsorption and the simultaneous electrostatic repulsion from p-SNF achieve the stable confinement of MB in the electrostatic nanocage of bp-SNF. In addition, the recognitive aptamer of CEA is covalently immobilized by the derivatization of amino groups on bp-SNF using glutaraldehyde (GA). In combination, stable and abundant immobilized probes and highly specific aptamer interfaces, the constructed sensor can be applied for the reagentless detection of CEA with high performance. 

## 2. Materials and Methods

### 2.1. Chemicals and Materials

Carcinoembryonic antigen (CEA), alpha fetoprotein (AFP), prostate-specific antigen (PSA), and cancer antigen 15-3 (CA15-3) were purchased from Beijing KEY-BIO Biotech Co., Ltd. (Beijing, China). C-reactive protein (CRP) was obtained from Nanjing Okay Biotechnology Co., Ltd. (Nanjing, China). Amino-modified CEA aptamer (5′-ATACAGCTTCAATT-NH_2_-3′) was purchased from Sangon Biotechnology Co., Ltd. (Shanghai, China). Cetyltrimethylammonium bromide (CTAB), ethyl orthosilicate (TEOS), potassium ferricyanide (K_3_[Fe(CN)_6_]), potassium ferrocyanide (K_4_[Fe(CN)_6_]), sodium dihydrogen phosphate dihydrate (NaH_2_PO_4_·2H_2_O), disodium hydrogen phosphate dodecahydrate (Na_2_HPO_4_·12H_2_O), potassium chloride (KCl), glutaraldehyde (GA), and methylene blue trihydrate (MB) were obtained from Shanghai Aladdin Biochemical Technology Co., Ltd. (Shanghai, China). Potassium hydrogen phthalate (KHP) and 3-aminopropyltriethoxysilane (APTES) were purchased from Shanghai McLean Reagent Co., Ltd. (Shanghai, China). Ruthenium (III) chloride (Ru(NH_3_)_6_Cl_3_) and bovine serum albumin (BSA) were obtained from Sigma-Aldrich Co., Ltd. (Shanghai, China). Sodium nitrate (NaNO_3_) and sodium hydroxide (NaOH) were obtained from Hangzhou Gaojing Fine Chemical Co., Ltd. (Hangzhou, China). Ethanol and concentrated hydrochloric acid were purchased from Hangzhou Shuanglin Chemical Reagent Co., Ltd. (Hangzhou, China). Phosphate buffer solution (PBS) was prepared by mixing NaH_2_PO_4_·2H_2_O and Na_2_HPO_4_·12H_2_O in a certain proportion. All chemicals and reagents in this experiment were analytically pure and had not been further purified. All aqueous solutions were prepared using ultrapure water (18.2 M Ω cm). Indium tin oxide (ITO) conductive glass electrode (square resistance < 17 Ω/square, thickness: 100 ± 20 nm) was purchased from Zhuhai Kaiwei Photoelectric Technology Co., Ltd. (Zhuhai, China). Before use, ITO electrode was immersed in NaOH (1 M) overnight. Then, the electrode was ultrasonically treated with acetone, ethanol, and ultrapure water, successively. The clean electrode was finally dried with N_2_.

### 2.2. Measurements and Instrumentations

The morphology of bp-SNF was observed using transmission electron microscopy (TEM, JEM-2100, JEOL, Tokyo, Japan) and scanning electron microscopy (SEM, SU8010, Hitachi, Tokyo, Japan). For the TEM investigation, bp-SNF was gently scraped from the surface of the ITO electrode and dispersed in ethanol. After ultrasonic treatment, the resulting solution was dropped onto the supporting copper mesh. All electrochemical tests were conducted at the Autolab electrochemical workstation (PGSTAT302N, Metrohm, Herisau, Switzerland). The three-electrode system was applied. Briefly, bare or modified ITO electrode acted as the working electrode, a platinum wire was the counter electrode, and an Ag/AgCl electrode (saturated with KCl) was applied as the reference electrode. The supporting electrolyte solution was KHP (0.05 M). The concentration of the two standard electrochemical probes including K_3_Fe(CN)_6_ and Ru(NH_3_)_6_Cl_3_ was 0.5 mM. The scanning rate for the cyclic voltammetry (CV) measurement was 50 mV/s. The parameters for the differential pulse voltammetry (DPV) measurement including step potential, pulse amplitude, pulse time, and interval time were 0.005 V, 0.05 V, 0.05 s, and 0.2 s, respectively. 

### 2.3. Preparation of n-SNF/ITO

The Stöber solution growth method was applied to grow n-SNF on a bare ITO electrode (1 cm × 1 cm) [46]. The precursor solution was first prepared. Briefly, CTAB (160 mg) was dissolved in a mixed solution containing ultrapure water (70 mL) and ethanol (30 mL) before ammonia (100 μL) and TEOS (80 μL) was added with stirring. Then, the cleaned ITO electrode was immersed in the precursor solution, and the reaction was kept at 60 °C for 24 h. Afterwards, the obtained electrode was thoroughly washed with a large amount of ultrapure water and aged overnight at 100 °C. The obtained electrode containing surfactant micelles (SM@n-SNF/ITO) was immersed in a 0.1 M HCl/ethanol solution and stirred for 5 min to remove the micelles. Finally, the n-SNF/ITO electrode was obtained.

### 2.4. Preparation of bp-SNF/ITO

The p-SNF layer was grown on the surface of n-SNF/ITO using an electrochemically assisted self-assembly method (EASA) [47,48,49]. CTAB (1.585 g) and 3-aminopropyltriethoxysilane (APTES, 0.318 mL) were added to the mixture of ethanol (20 mL) and NaNO_3_ (0.1 M, pH = 2.6, 20 mL). After the pH of the solution was adjusted to 2.97 using HCl (6 M), TEOS (2.372 mL) was added and stirred at room temperature for 2.5 h. When n-SNF/ITO electrode was placed in the above precursor solution, a constant current density (−0.70 mA cm^−2^) was applied for 15 s. Then, the obtained electrode was rapidly rinsed with ultrapure water and aged overnight at 100 °C. After removal of SM, bp-SNF/ITO electrode with an electrostatic nanocage was obtained.

### 2.5. Immobilization of MB and an Aptamer on bp-SNF/ITO Electrode

To enrich MB, bp-SNF/ITO electrode was immersed in MB solution (0.01 M in PBS, pH = 7.4) with stirring for 10 min. Then, the electrode was thoroughly rinsed with ultrapure water to obtain MB@bp-SNF/ITO electrode. Using GA as a cross-linking agent, the aptamer was covalently immobilized on the external surface of MB@bp-SNF/ITO electrode. Briefly, MB@bp-SNF/ITO electrode was immersed in GA (5%, 50 μL) and incubated at 37 °C for 1 h in darkness. After rinsing the unreacted GA with PBS (0.01 M, pH = 7.4), the GA/MB@bp-SNF/ITO electrode was immersed in CEA aptamer (0.1 μM) and incubated at 4 °C for 2 h to obtain Apt/GA/MB@bp-SNF/ITO electrode. After washing off the residual CEA aptamer, non-specific sites were sealed with BSA (0.5 wt%) to obtain an aptamer sensor.

### 2.6. Electrochemical Detection of CEA

For the electrochemical detection of CEA, PBS (0.01 M, pH = 7.4) was applied as the electrolyte. The constructed aptamer sensor was incubated with different concentrations of CEA at 4 °C for 1.5 h. The peak redox currents of MB before and after incubation with CEA were measured using DPV. For real sample analysis, CEA in human serum of a healthy woman was detected after the sample was diluted using PBS (0.01 M, pH = 7.4) by a factor of 50. The detection of CEA in fetal bovine serum was also performed using the standard addition method. Briefly, artificial CEA was added into the serum samples. The samples were diluted by a factor of 50 with PBS (0.01 M, pH = 7.4) before electrochemical detection using the fabricated biosensor. All measurements were performed three times to obtain the relative standard deviation (RSD).

## 3. Results and Discussion

### 3.1. Construction of Electrochemical Aptamer Sensor with Integrated Electrochemical Probe

As the tumor biomarker CEA, itself, does not possess redox activity, electrochemical probes are, thus, required for the detection. Commonly, the binding between CEA and its recognitive ligand changes the signal of the electrochemical probe, leading to the electrochemical detection of CEA. Two sensing modes are usually included. One is to add a free electrochemical probe to the solution, and the other is to immobilize the electrochemical probe on the electrode surface. Compared to the former detection mode, the latter mode with integrated probes has a simple detection process, which can achieve reagentless detection and avoid the impact of free probes on the detection solution. The stable immobilization of a large number of electrochemical probes on the electrode surface is the key for reagent-free electrochemical detection of CEA.

**Figure 1:**
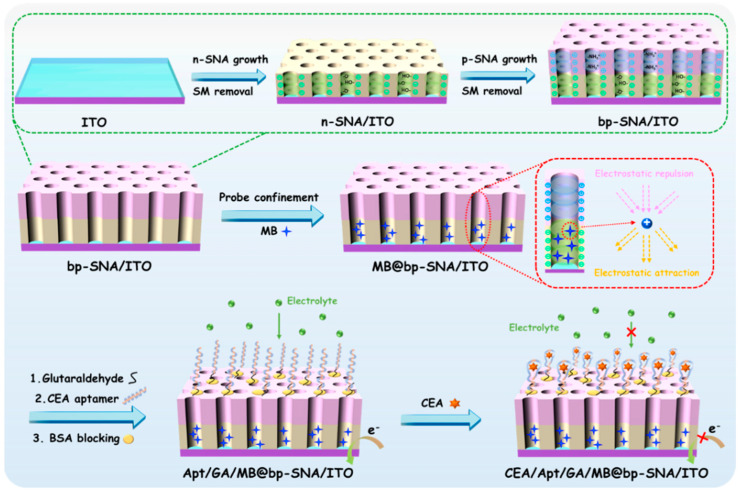
Schematic illustration for the fabrication of electrochemical aptamer sensor based on MB@bp-SNF/ITO electrode and reagentless detection of CEA.

Figure 1 illustrates the construction of the probe-integrated aptamer immunosensor. As shown, a bipolar film (bp-SNF) with asymmetric surface charges is first prepared by sequentially growing two layers of SNF on the surface of indium tin oxide (ITO) electrodes. These two layers of SNF are grown using different precursors, which in turn lead to different surface charge properties. Briefly, the inner SNF has a negative charge, which is recorded as n-SNF. The outer SNF has a positive charge and is designated as p-SNF. For the growth of SNF, the key is the hydrolysis and condensation of siloxanes in the presence of cationic surfactant micelles (SM). The Stöber solution growth method is applied for the growth of n-SNF on ITO, which regulates the hydrolysis and polycondensation of siloxane in ammonia–ethanol medium. Although the growth time is long (24 h), a large area of SNF can be prepared at one time. SM in nanochannels can easily be removed by immersing the electrode in HCl-ethanol solution. Subsequently, the electrochemically assisted self-assembly (EASA) method, which can easily grow SNF in a few seconds, is used to grow p-SNF with positive charges and rich surface amino groups on n-SNF/ITO by introducing a siloxane precursor containing amino groups. Briefly, when water is reduced at negative potential, the in-situ-generated hydroxyl ions induce the self-assembly of SM on the electrode surface, catalyzing the hydrolysis and polycondensation of siloxanes. After SM is removed, the bp-SNF/ITO electrode with an electrostatic nanocage array is obtained.

As illustrated in Figure 1, the commonly used electrochemical probe methylene blue (MB) is immobilized on bp-SNF. Then, a bifunctional cross-linking agent glutaraldehyde (GA) is introduced and reacts with the amino group on the outer surface of bp-SNF to produce an active surface with aldehyde groups. The biological interface is further fabricated through the covalent linkage of a recognitive aptamer followed by blocking non-specific sites with bovine serum albumin (BSA). The binding of CEA with the adapter on the sensing interface causes a decrease in the electrochemical signal of MB, achieving reagentless electrochemical detection.

### 3.2. Characterization of bp-SNF Modified Electrode

**Figure 2:**
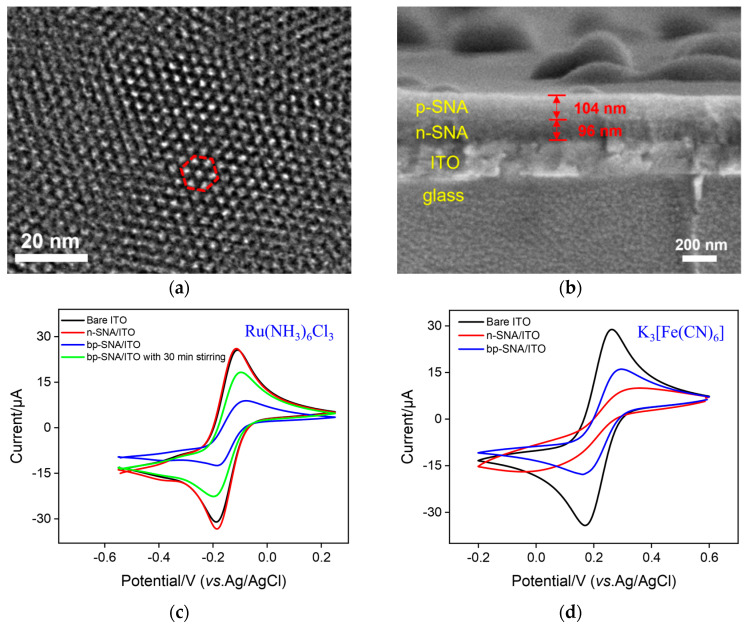
(**a**) Top-view TEM image of bp-SNF. (**b**) Cross-sectional SEM image of bp-SNF/ITO. The red region indicates the hexagonal structure of pores. (**c**) CV curves obtained on bare ITO, n-SNF/ITO, and bp-SNF/ITO with or without 30 min stirring in KHP (0.05 M, pH = 4) containing Ru(NH_3_)_6_Cl_3_ (0.5 mM). (**d**) CV curves obtained on bare ITO, n-SNF/ITO, and bp-SNF/ITO in KHP (0.05 M, pH = 4) containing K_3_[Fe(CN)_6_] (0.5 mM).

The morphology of bp-SNF was characterized by transmission electron microscopy (TEM) and scanning electron microscopy (SEM). As shown in Figure 2a, the top-view TEM image of bp-SNF reveals a porous film with ordered pores, which are stacked into a hexagonal structure. Within the observed range, the film has no cracks. The cross-sectional SEM image of bp-SNF/ITO indicates a four-layered structure, which contains a glass layer, ITO layer, n-SNF, and p-SNF, from bottom to top (Figure 2b). The thicknesses of the n-SNF and p-SNF layers are about 96 nm and 104 nm, respectively. The silicon balls on the surface can easily be removed using adhesive tape.

The diffusion performance of small molecules on the bp-SNF/ITO electrode was investigated using standard electrochemical probes including a cationic probe (Ru(NH_3_)_6_^3+^) and an anionic probe (Fe(CN)_6_^3−^). For comparison, the electrochemical signals of these two probes on bare ITO and n-SNF/ITO electrodes were also investigated. For the Ru(NH_3_)_6_^3+^ probe, the n-SNF/ITO electrode shows similar electrochemical signals in comparison with the bare ITO (Figure 2c), while the signal of Fe(CN)_6_^3−^ on the n-SNF/ITO electrode is significantly lower than that on the bare ITO electrode (Figure 2d). This is attributed to the charge selectivity of ultrasmall nanochannels. Specifically, n-SNF is rich in silanol groups (Si-OH, p*K*_a_ ~ 2), leading to a negatively charged surface when Si-OH ionizes. Thus, n-SNF exhibits significant electrostatic enrichment towards Ru(NH_3_)_6_^3+^ probes, while exhibiting electrostatic repulsion to Fe(CN)_6_^3−^. At the same time, p-SNF is positively charged due to amino groups on the surface, which display electrostatic repulsion towards Ru(NH_3_)_6_^3+^ and electrostatic attraction towards Fe(CN)_6_^3−^, resulting in a lower peak current for Ru(NH_3_)_6_^3+^ and a higher signal for Fe(CN)_6_^3−^ on the bp-SNF/ITO electrode. When the Ru(NH_3_)_6_^3+^ solution is stirred, its signal on the bp-SNF/ITO electrode significantly increases (Figure 2c). Thus, Ru(NH_3_)_6_^3+^ ions in the solution can be pushed to break through the outer barrier of the bipolar film and enter the inner SNF.

### 3.3. Stable Confinement of MB on bp-SNF/ITO

**Figure 3:**
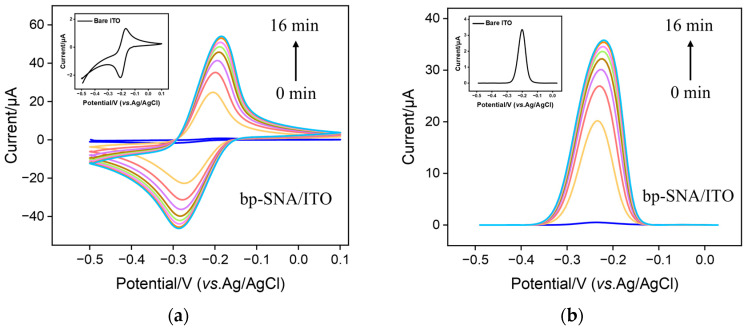

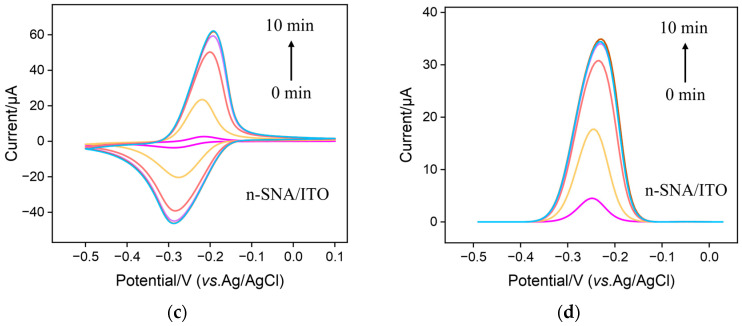
CV (**a**,**c**) and DPV (**b**,**d**) curves obtained from bp-SNF/ITO (**a**,**b**) or n-SNF/ITO (**c**,**d**) electrode in MB solution (10 μM) when the solution is stirred for different amounts of time. Insets in a and b are the corresponding curves obtained from ITO electrode.

As cationic probes can break through the barrier of bp-SNF with stirring, the immobilization of MB on the bp-SNF/ITO electrode through the electrostatic enrichment by the inner n-SNF was investigated. Figure 3 a and b display the cyclic voltammetry (CV) and differential pulse voltammetry (DPV) curves obtained from the bp-SNF/ITO electrode in MB solution when the solution was stirred for different amounts of time. As seen, the signal of MB on the electrode significantly increases as the stirring time increases. After stirring for 14 min, the signal reaches the plateau. The highest peak current is significantly higher than that obtained from the bare ITO electrode (insets in Figure 3a,b). For comparison, the corresponding CV and DPV curves obtained from the n-SNF/ITO electrodes are given in Figure 3c,d. An increase in stirring time will also lead to an increased MB signal, which stabilizes after 8 min. Although n-SNF/ITO can enrich MB faster, the peak current of MB after enrichment stabilization is close to that obtained from the bp-SNF/ITO electrode. This indicates that the amount of MB enriched by bp-SNF is similar with that by n-SNF.

**Figure 4:**
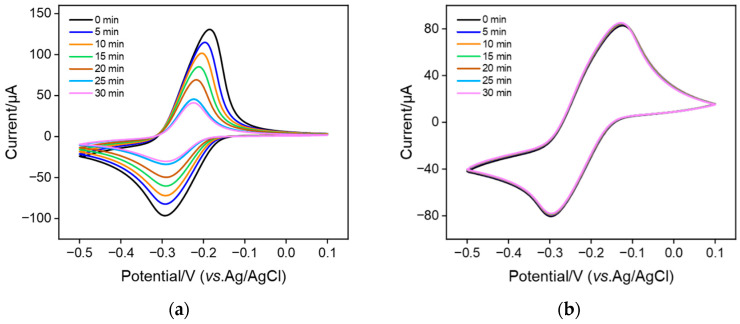
CV curves obtained from (**a**) MB@n-SNF/ITO and (**b**) MB@bp-SNF/ITO electrodes in 0.01 M PBS (pH 7.4) at different times. CV measurement is performed at 5-min intervals.

To investigate the stability of MB confined in nanochannels, MB@bp-SNF/ITO and MB@n-SNF/ITO electrodes were obtained when bp-SNF/ITO and n-SNF/ITO electrodes were immersed in MB solution (0.1 mM) with stirring for 10 min. The electrochemical signal of MB on the MB@bp-SNF/ITO or MB@n-SNF/ITO electrodes in PBS at different times was measured (Figure 4). As seen, the signal of MB on MB@n-SNF/ITO significantly decreases (Figure 4a). Only about 30% of the initial signal is observed after 30 min. However, the redox current of MB obtained on the MB@bp-SNF/ITO electrode remains essentially unchanged after 30 min (Figure 4b). This phenomenon indicates that the stability of MB immobilized on the monolayer n-SNF is low, indicating the easy diffusion of MB in the electrolyte. On the contrary, MB confined on bp-SNF has a high stability. This is attributed to the dual electrostatic effect by the electrostatic nanocages of bp-SNF. Briefly, MB confined on the inner n-SNF is simultaneously attracted to n-SNF and repelled by the outer p-SNF. This dual electrostatic barrier makes it difficult for MB to escape from the inner nanochannel, thus achieving high stability.

### 3.4. Fabrication of Aptamer Sensor for Electrochemical Detection of CEA

**Figure 5:**
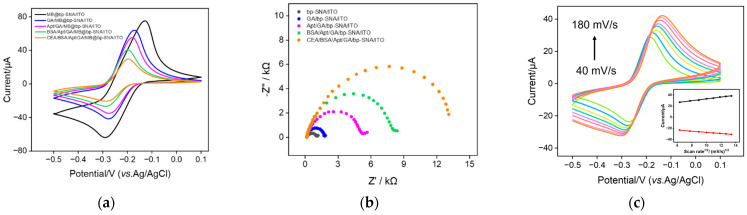
CV (**a**) and EIS (**b**) curves obtained from different electrodes in the fabrication of the aptamer sensor. The electrolyte for CV measurements is PBS (0.01 M, pH 7.4). The solution for the EIS test is 0.1 M KCl containing 2.5 mM Fe(CN)_6_^3−/4−^. (**c**) CV curves of the CEA/BSA/Apt/GA/MB@bp-SNA/ITO electrode in 0.01 M PBS (pH 7.4) at various scan rates. Inset is the dependence of anodic (red line) and cathodic (black line) peak currents on the square root of scan rate.

The feasibility for construction of the aptamer sensor was investigated using CV and electrochemical impedance spectroscopy (EIS). As shown in Figure 1b, the amino groups on the outer surface of MB/bp-SNA/ITO are derivatized using GA (GA/MB/bp-SNA/ITO), followed by covalent immobilization of the amino-modified CEA aptamer (Apt/GA/MB/bp-SNA/ITO) and BSA blocking of non-specific sites (BSA/Apt/GA/MB/bp-SNA/ITO). Thus, the aptamer sensor is obtained. As shown in Figure 5a, the GA derivatization, aptamer immobilization, and non-specific blocking all lead to a gradual decrease in the peak current of MB because of the increased surface resistance of the electrode. When the constructed aptamer sensor is incubated with CEA, the aptamer specifically binds to CEA, resulting in a further decrease in the redox peak current and an increase in the peak-to-peak difference, owing to the formation of an immunocomplex. In the EIS spectrum of Figure 5b, the charge-transfer resistance of the electrode increases with the gradual modification of the electrode mentioned above. This is consistent with the current variation in the CV curves. Figure 5c gives the CV curves of the 1 ng/mL CEA captured by the aptamer sensor in 0.01 M PBS (pH 7.4) at different scan rates. When scan rates increase, both anodic and cathodic peak currents increase. Additionally, the linear relationship between peak currents and the square root of the scan rate was observed (Ip_a_ = 1.64 v^1/2^ + 16.54, R^2^ = 0.992; Ip_c_ = −1.05 v^1/2^ − 16.85, R^2^ = 0.993), illustrating a diffusion-controlled process. This phenomenon further proves that the MB probe in bp-SNA nanochannels is still diffusive.

### 3.5. Electrochemical Detection of CEA Using the Fabricated Aptamer Sensor

**Figure 6:**
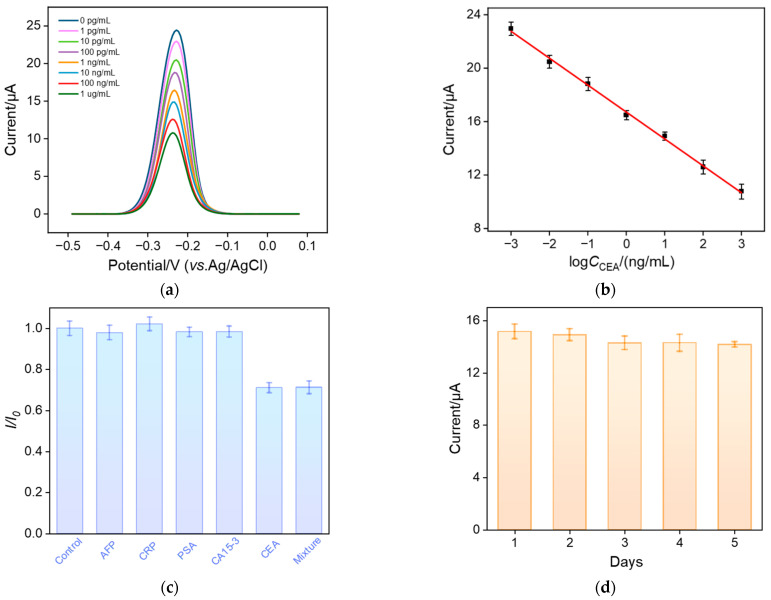
(**a**) DPV curves obtained using the aptamer sensor in the presence of different concentrations of CEA (1 pg/mL ~ 1 μg/mL). (**b**) The corresponding calibration curve. (**c**) Relative ratio (I/I_0_) of peak current before (I_0_) and after (I) incubation with buffer (control), AFP, CRP, PSA, CA15-3, CEA, and their mixture. The concentrations of AFP, CRP, and PSA are 10 ng/mL, and the concentration of CA15-3 is 10 μU/mL. The concentration of the target CEA is 1 ng/mL. (**d**) Stability of the aptamer sensor. Error bars represent the standard deviations of three measurements.

The performance of the constructed aptamer sensor for the electrochemical detection of CEA was investigated. Figure 6a shows the DPV response obtained from the BSA/Apt/GA/MB@bp-SNA/ITO electrode in the presence of different concentrations of CEA. As seen, the anodic peak current of MB on the electrode significantly decreases as the concentration of CEA increases, which is attributed to the increase in surface resistance caused by the combination of CEA and the aptamer. As shown in Figure 6b, the peak current of MB (I) exhibits a good linear relationship with the logarithmic value of CEA concentration (logC_CEA_) within the range of 1 pg/mL to 1 μg/mL (I = −2.01 logC_CEA_ + 16.72, R^2^ = 0.998). The limit of detection (LOD) was calculated to be 0.22 pg/mL based on a signal-to-noise ratio of three (S/N = 3). Table 1 summarizes the analytical performance of various electrochemical methods for the detection of CEA [27,29,50,51,52,53]. As seen, the LOD of the fabricated aptamer is only higher than that obtained from the immunosensor fabricated by the immobilization of the CEA antibody (Ab) on cuprous-sulfide–palladium–copper-oxide-modified glassy carbon electrode (Ab/Cu_2_S/Pd/CuO/GCE) [50]. In addition, most sensors require time-consuming and laborious procedures, while the developed aptamer sensor does not need the synthesis of complex nanomaterials.

### 3.6. Selectivity, Reproductivity, and Stability of the Fabricated Aptamer Sensor

The selectivity of the sensor was examined by incubating the constructed aptamer sensor with CEA or other single- or mixed-tumor biomarkers. Specifically, the BSA/Apt/GA/MB@bp-SNF/ITO electrode was incubated with alpha fetoprotein (AFP), C-reactive protein (CRP), prostate-specific antigen (PSA), cancer antigen 15-3 (CA15-3), CEA, and their mixtures. As shown in Figure 6c, the redox peak current of MB on the aptamer sensor significantly decreases only after the sensor is incubated with CEA or a mixture containing the five antigens. This phenomenon indicates that the fabricated aptamer sensor has high selectivity, which is attributed to the highly specific recognition between the aptamer and CEA. To investigate its reproducibility, five aptamer sensors were constructed in parallel. The relative standard deviation (RSD) for the detection of CEA (10 ng/mL) was 2.4%, suggesting high reproducibility. The storage stability of the aptamer sensor was also investigated. The constructed aptamer sensor was stored at 4 °C for 5 days. When CEA (10 ng/mL) was detected, the peak current after 5-day storage remained at 93.5% of the initial measurement, demonstrating high storage stability (Figure 6d). The simple and repeatable preparation method, rigid/non-swelling structure, and high biocompatibility of SNF result in the high reproducibility and stability of the sensor.

### 3.7. Real Sample Analysis

The application of the fabricated sensor for the detection of CEA in a real sample was investigated. The standard addition method was applied to evaluate the detection performance of the aptamer sensor. Briefly, fetal bovine serum without CEA was applied as the complex matrix and different artificial concentrations of CEA were added. Before testing, fetal bovine serum with added CEA was diluted with PBS (0.01 M, pH 7.4) by a factor of 50. As shown in Table 2, satisfactory recovery ranging from 98.9 to 103% was obtained with low relative standard deviation (RSD < 3.5%). Therefore, this aptamer sensor has great potential for the sensitive detection of CEA in clinical biological samples. 

## 4. Conclusions

In summary, an aptamer sensor with an integrated electrochemical probe in bipolar SAN nanochannels was developed for the label-free electrochemical detection of CEA. A bp-SNF with a bilayer nanochannel structure and asymmetric charges was modified on the supporting ITO electrode. By stirring, the positively charged electrochemical probe MB can break through the electrostatic repulsion from the outer p-SNF and enter the inner n-SNF for electrostatic enrichment. The dual electrostatic effect enables MB to be stably confined within the electrostatic nanocages of bp-SNF. The biological recognitive interface was then fabricated through the covalent immobilization of the aptamer on the outer surface of bp-SNF. The constructed aptamer sensor has high selectivity and can be used for the sensitive electrochemical detection of CEA. Due to the large and stable immobilization of electrochemical probes by bp-SNF and the recognitive ability of the aptamer, the constructed sensor exhibits high sensitivity and good reproducibility for CEA detection. The probe-integrated aptamer sensor constructed in this work has great potential in reagent-free electrochemical detection of tumor biomarkers.

## Figures and Tables

**Table 1:** Comparison of the analytical performance of different modified electrodes for the determination of CEA.

Electrode	Method	Linear Range (ng/mL)	LOD (pg/mL)	Sample	Preparation Time of Sensor * (h)	Ref.
CEA/BSA/Ab/Cu_2_S/Pd/CuO/GCE	I-t	10^−4^–100	0.03311	Human serum	13.3	[50]
PDA@Gr/Pd-PtNDs/ hemin/G4/CEA/Apt1/PDA@Gr/GCE	DPV	0.05–10^3^	6.3	Human serum	15.5	[27]
CEA/BSA/Ab/Au/ZnMn_2_O_4_@rGO/GCE	DPV	0.01–50	1.93	Human serum	>27	[51]
hybrid DNA/CEA-H1/BSA/MCH/H2/Au	DPV	0.01–100	0.84	Human serum	15	[29]
NCMTs@Fe_3_O_4_@Cusilicate/ConA/CEA/AuNCs-aptamer	DPV	0.03–6	5.38	Human serum	>16.5	[52]
CEA/MCH/aptamer/AuNPs/PPy	EIS	0.1–10^3^	33	Fetal bovine serum	>14	[53]
CEA/BSA/Apt/GA/MB@bp-SNA/ITO	DPV	10^−3^–10^3^	0.22	Fetal bovine serum	5.2	This work

Ab, antibody; Cu_2_S, cuprous sulfide; Pd, palladium; CuO, copper oxide; GCE, glass carbon electrode; PDA@Gr, polydopamine functional graphene; Pd-PtNDs, Pd-Pt nanodendrites; hemin/G4, hemin/G-quadruplex; ZnMn_2_O_4_@rGO, ZnMn_2_O_4_@ reduced graphene oxide; H1, hairpin probe 1; MCH, 6-mercapto-1-hexanol; H2, hairpin probe 2; NCMTs@Fe_3_O_4_@Cusilicate, copper silicate integrated with nitrogen-doped magnetic carbon microtubes; ConA, concanavalin A; AuNCs, gold nanoclusters; PPy, polypyrrole; DPV, differential pulse voltammetry; EIS, electrochemical impedance spectroscopy. * Accurate preparation time of sensors were calculated based on experimental procedures.

**Table 2:** Determination of CEA in fetal bovine serum using the fabricated aptamer immunosensor.

Sample	Added ^a^ (ng/mL)	Found (ng/mL)	Recovery (%)	RSD (%, n = 3)
serum	0.0100	0.00989	98.9	3.5
	1.00	1.03	103	2.5
	100	98.9	98.9	3.1

^a^ the fetal bovine serum is diluted by a factor of 50 using PBS (0.01 M, pH 7.4). The indicated concentration of CEA is obtained after the dilution.

## Data Availability

The data presented in this study are available upon request from the corresponding author.
